# Cost-Effectiveness and Burden of Disease for Adjuvanted Quadrivalent Influenza Vaccines Compared to High-Dose Quadrivalent Influenza Vaccines in Elderly Patients in Spain

**DOI:** 10.3390/vaccines10020176

**Published:** 2022-01-23

**Authors:** Jesús Ruiz-Aragón, Sergio Márquez-Peláez, Ray Gani, Piedad Alvarez, Richard Guerrero-Luduena

**Affiliations:** 1 Hospital de La Línea, 11300 La Línea de la Concepción, Spain; reducido@hotmail.com; 2 Department of Economics, Faculty of Business. Universidad Pablo de Olavide, 41013 Sevilla, Spain; smarpel@upo.es; 3 Evidence, Modeling, and Synthesis, Evidera, London W6 8BJ, UK; richard.guerrero-luduena@evidera.com; 4 Evidence, Modeling, and Synthesis, Evidera, Waltham, MA 02451, USA; piedad.alvarez@evidera.com

**Keywords:** influenza, vaccination, Spain, cost-effectiveness, adjuvanted, high dose, burden of illness

## Abstract

Influenza is a contagious respiratory disease that causes severe illness and death, particularly in elderly populations. Two enhanced formulations of quadrivalent influenza vaccine (QIV) are available in Spain. Adjuvanted QIV (aQIV) is available for those aged 65+ and high-dose QIV (HD-QIV) for those aged 60+. In this study, we used a health economic model to assess the costs and outcomes associated with using aQIV or HD-QIV in subjects aged 65+. Using aQIV instead of HD-QIV to vaccinate an estimated 5,126,343 elderly people results in reductions of 5405 symptomatic cases, 760 primary care visits, 171 emergency room visits, 442 hospitalizations, and 26 deaths in Spain each year. Life-years (LYs) and quality-adjusted LYs (QALYs) increases by 260 and 206, respectively, each year. Savings from a direct medical payer perspective are EUR 63.6 million, driven by the lower aQIV vaccine price and a minor advantage in effectiveness. From a societal perspective, savings increase to EUR 64.2 million. Results are supported by scenario and sensitivity analyses. When vaccine prices are assumed equal, aQIV remains dominant compared to HD-QIV. Potential savings are estimated at over EUR 61 million in vaccine costs alone. Therefore, aQIV provides a highly cost-effective alternative to HD-QIV for people aged 65+ in Spain.

## 1. Introduction

Seasonal influenza is an acute respiratory infection caused by influenza viruses, which circulate in all parts of the world. It is characterized by a sudden onset of fever, cough, headache, muscle and joint pain, severe malaise, sore throat, and a runny nose. Whilst most people quickly recover without requiring medical attention, worldwide influenza is estimated to cause three to five million cases of severe illness and 290,000 to 650,000 respiratory deaths each year [1]. An analysis of the EuroMOMO network estimated 152,000 deaths (150,000 to 155,000) in the 2017/2018 influenza season in Europe [2].

Influenza spreads rapidly during the winter months resulting in epidemics that lead to high demand for healthcare resources and substantial economic burden. The average incidence in Spain is estimated at 2000 cases per 100,000 inhabitants, with associated costs due to primary care, hospital care, treatments, and absences from work of EUR 1 billion per year [3]. A substantial proportion of this burden is associated with patients aged 65 years and older (65+) and, as a consequence, many countries, including Spain, recommend routine annual vaccination against influenza in people aged 65+ [4].

Four types of influenza viruses (A to D) are currently in circulation, with influenza A and B as the main ones responsible for seasonal epidemics in humans. Influenza A is classified into subtypes based on combinations of hemagglutinin (HA) and neuraminidase (NA). Currently circulating influenza A virus subtypes are A/H1N1 (also known as A/H1N1 pdm09) and A/H3N2. The commonly circulating strains for influenza B are B/Yamagata or B/Victoria [5]. Quadrivalent influenza vaccines (QIVs) are designed to provide protection against all four of these subtypes [1].

There are a number of QIVs available in Spain and two enhanced QIVs that have been recently licensed [6,7]. Adjuvanted QIV (aQIV), available for people aged 65+, combines MF59 adjuvant (an oil-in-water emulsion of squalene oil) and a standard dose of antigen, and is designed to produce stronger, broader, and longer immune responses against the selected influenza vaccine strains [8]. HD-QIV contains a higher concentration of antigen than the standard-dose influenza vaccines and is designed to produce stronger immune responses against the selected influenza vaccine strains [9]. Both have been developed to provide improved protection among older age groups in whom immune responses with regular standard-dose QIVs can be suboptimal. The objective of this study was to determine the cost-effectiveness and burden of disease associated with vaccinating the population aged 65+ with either aQIV or HD-QIV in Spain.

## 2. Materials and Methods

### 2.1. Model Structure

**Figure 1:**
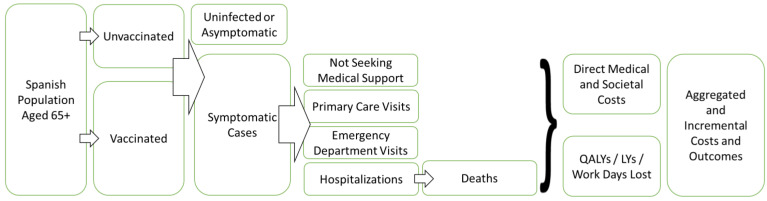
Schematic of the health-economic model. Abbreviations: LYs = life years; QALYs = quality-adjusted life years.

A health economic model simulating the costs, benefits, and burden of disease for the Spanish population aged 65+ vaccinated with either aQIV or HD-QIV over a single influenza season was developed. The model was based on the static, decision-tree model developed by Ruiz-Aragón et al. for the Spanish setting [10]. This structure has been used extensively in other influenza cost-effectiveness analyses [11,12,13,14,15], and the analysis was designed in line with Spanish best practices for health economic modeling [16]. A schematic of the economic is shown in Figure 1.

In the model, the Spanish population aged 65+ can be either vaccinated or unvaccinated. Vaccinated people in one comparator arm receive aQIV and in the other arm they receive HD-QIV. People from both the vaccinated and unvaccinated populations then, over the course of the one-year time-horizon (which represents one influenza season), enter one of the following disease states: uninfected or asymptomatic; symptomatic cases not seeking medical support; or symptomatic cases requiring either a primary care visit, emergency department visit, or hospitalization. Patients hospitalized then have a probability of death. Each state has a fixed cost and disutility associated with it. Costs and outcomes are finally aggregated across the different states to calculate the totals for each cohort. Two cost perspectives are included: direct medical payer and societal. All costs and outcomes are calculated for an entire influenza season, except for productivity loss due to death and quality-adjusted life year (QALY) loss due to death. These are calculated over a lifetime horizon and discounted at 3% per year, following Spanish cost-effectiveness guidelines [16]. This discount rate is applied to both costs and QALYs.

### 2.2. Epidemiology

Vaccine coverage, population size, and life expectancy for the 65+ population was taken from national 2021 Spanish statistics [17,18,19]. Vaccine coverage was 54.7% [17], life expectancy was 9.8 years [18], and the population size was 9,371,743 [19].

### 2.3. Rates of Clinical Outcomes

The rates per 100,000 for the different clinical outcomes are shown in Table 1. These are based on the influenza seasons from 2017 to 2018, 2018 to 2019, and 2019 to 2020. Incidence of clinically reported influenza cases in the Spanish population was taken from surveillance reports from the sentinel general practitioners of the Sistema Centinela de Vigilancia de Gripe in Spain (ScVGE) [20,21,22]. Patients were split into those that visit a primary care physician (81.67%) and those that visit an emergency department (18.33%) [23]. The distribution of hospitalizations were also taken from Spanish public reports [20,21,22]. The death rate was based on the calculated mortality rates of 6% per hospitalization from Crepey et al. [24]. The average across these three influenza seasons were used to estimate baseline incidence rates in the model base case.

The outcomes in the model from the Spanish sentinel surveys are based on a mix of vaccinated and unvaccinated people. We assume that this population is vaccinated with standard-dose QIV (SD-QIV). We assume the relative vaccine efficacy for HD-QIV vs. SD-QIV is 24% [25,26] which we use in the base-case for the model.

### 2.4. Vaccine Effectiveness

Studies reporting the relative vaccine effectiveness of adjuvanted trivalent and quadrivalent influenza vaccines (aTIV or aQIV) compared to high-dose trivalent and quadrivalent influenza vaccines (HD-TIV and HD-QIV) for the prevention of influenza-related hospitalizations (or composite outcomes including influenza-related hospital admissions) were identified from a systematic review that covered publications from 1997 (first licensure of aTIV) to 15 July, 2020 [27]. Additionally, a targeted non-systematic review was conducted by a single reviewer by searching in PubMed in July 2021 to identify potential additional relevant studies published between July 2020 and July 2021. The PRISMA checklist for the additional searches is provided in the Supplementary Table S1 Information. Data were extracted into a structured data template that captured the study design, season of study, intervention, comparator, outcome definitions, and effect estimates/confidence intervals. The quadrivalent formulations for the adjuvanted and high-dose seasonal influenza vaccines were first available during the 2020–2021 influenza season and therefore only publications evaluating the relative vaccine effectiveness of their trivalent predecessors were identified in the review. It is assumed that the relative vaccine effectiveness of the two quadrivalent vaccines would be equivalent to the relative vaccine effectiveness of the two trivalent vaccines.

**Figure 2:**
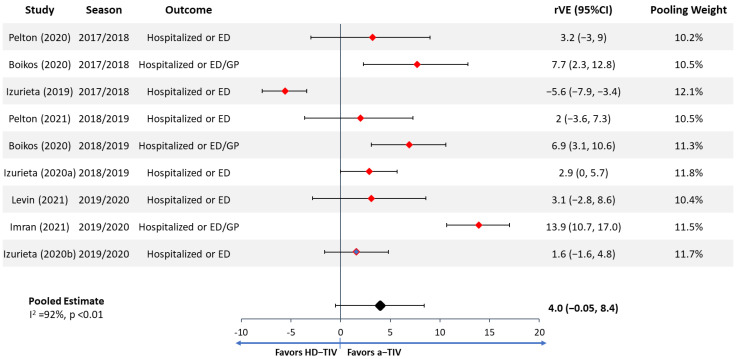
Meta-analysis of effect estimates from identified studies reporting the relative vaccine effectiveness of aTIV vs. HD-TIV for prevention of influenza-related hospitalizations (or composite outcomes including influenza-related hospital admissions). Study pooling weights were calculated based on DerSimonian and Laird random-effects meta-analysis [41]. Abbreviations: CI = confidence interval; ED = emergency department; GP = general practitioner; rVE = relative vaccine effectiveness.

A total of eight publications were included in the meta-analysis, one of which reported separate effect estimates for two seasons and was therefore included twice in the meta-analysis. Four studies [28,29,30,31] were identified from the published systematic review. The remaining four studies/effect estimates [32,33,34,35] were identified via the targeted review, two of which were not yet published as of the time of the analysis. As the relative vaccine effectiveness of aTIV vs. HD-TIV may be expected to vary based on the characteristics of the study (e.g., influenza season, population included, outcome definition, etc.), a random-effects model was used for the meta-analysis [36]. The meta-analysis was conducted using the R [37] package *meta* [38] in R 4.0.2. The forest plots of the data and relative effectiveness estimates are shown in Figure 2.

Identified publications were all retrospective cohort studies and reported outcomes based on incidence rate ratios (IRR) and odds ratios (OR), which were included in the same meta-analysis based on the assumption that ORs would approximate IRRs due to influenza hospitalization being rare outcome. Vaccine effectiveness was back calculated to IRR/OR for synthesis and then converted back to vaccine effectiveness. Identified publications reported relevant data for the three US influenza seasons from 2017 to 2020. During those three seasons, the trivalent formulation of influenza vaccines contained B-Victoria [39] and not B-Yamagata. During the 2017-2018 influenza season in the United States [40], approximately 24% of circulating viruses among patients aged ≥65 were B-Yamagata while the same was true for only about 1% of the circulating viruses during the 2018–2019 and 2019–2020 seasons.

The pooled estimated of the relative vaccine effectiveness of aTIV compared to HD-TIV was 4.0% (95% CI: −0.05 to 8.4), indicating that the point estimate favored aTIV over HD-TIV for prevention of influenza-related hospitalizations (or composite outcomes including influenza-related hospital admissions), but the difference was not statistically significant. There was high heterogeneity (I^2^ = 92%, *p* <0.01) due to variability in effect estimates between studies. Between study heterogeneity may be due to differences in study design/outcome selection, characteristics of the underlying study populations, and characteristics of the influenza season studied.

### 2.5. Utilities

Health-related quality of life (HRQoL) for clinical events was taken from Hollmann et al. [42]. These values were from a longitudinal study of Spanish patients from major hospitals. Patients reported their HRQoL using the EQ-5D-3L instrument for the influenza period and the week before. The disutility was calculated as the difference between EQ-5D-3L prior to the influenza episode and during it. Estimated duration of disutility for inpatients and outpatients was 21 and seven days, respectively. Disutility for inpatients was 0.60 and for outpatients was 0.33. Disutility for symptomatic cases was from Dolk et al. and estimated at 0.32 for seven days [43]. Baseline utility for the cohort of people aged 65+ was 0.65 [23].

### 2.6. Costs

The model was run using tender prices for vaccines, which were EUR 13 for aQIV and EUR 25 for HD-QIV [44]. These were used in the base case, with a scenario analysis using the list prices, which are EUR 23 for aQIV and EUR 32 for HD-QIV [45]. The resource unit costs were collected from official Spanish sources, including three bulletins of the Autonomous Communities: Andalucía, Murcia, and País Vasco. The middle value of them was selected for the model, with the cost of a primary-care physician visits at EUR 59 and emergency department visits at EUR 183 [46,47,48]. The hospitalization weighted average cost was calculated for relevant complications from 2019 APR-DRG statistical data published by the ministry of health and inflated to 2021 euros, and also included an intensive care unit stay for 9 days for 7.5% of admissions, at EUR 4467 [47,49]. All costs are for 2021. Patients with symptomatic disease who did not attend a primary care physician visit, emergency department visits or have an in-patient hospitalization were conservatively assumed to have no public payer or societal costs. A comedication cost of EUR 3.21 for the primary care visit was taken from the publication of Perez-Rubio and Eiros [50]. Administration and transportation costs were not included as they are expected to be the same across vaccines.

The societal perspective includes productivity losses due to direct illness, calculated using the discounted human capital approach and based on working days lost multiplied by the probability of being employed [51]. This was 1.2% for those aged 65 to 69 years and 0.3% for those aged 70+ [52]. Productivity loss per hour was EUR 17.44 [53]. The time spent caring for influenza patients at 5.4% for those aged 65 to 69 years and 14% for those aged 70+ was also included [54]. Productivity losses were assumed to be five working days for outpatients and 15 working days for inpatients [10].

### 2.7. Analysis

The outputs from the model include burden of illness, economic cost, and incremental analysis. The burden of illness outcomes included the number of symptomatic cases, primary care visits, emergency department visits, hospitalizations, and deaths when aQIV or HD-QIV is used to vaccinate the population aged 65+. Public payer costs and discounted societal costs were calculated as well as total discounted QALYs. The public payer costs were not discounted as they were only calculated over one year, whereas societal costs and QALY losses due to death were calculated based on life expectancy and discounted accordingly. Incremental cost-effectiveness ratios (ICERs) were calculated for aQIV vs. HD-QIV from a direct medical payer and societal perspective.

A series of scenario analyses were conducted to test the impact of the model assumptions on the ICERs. The impact of input uncertainty was evaluated through one-way deterministic sensitivity analysis (DSA). In addition, a probabilistic sensitivity analysis (PSA) was conducted by varying parameters based on their confidence intervals during 10,000 iterations of the model to assess the effect of uncertainty on the ICERs. The ICERs were compared against a willingness-to-pay threshold of EUR 25,000 per QALY gained. This is the willingness-to-pay threshold recently identified as the range used by the National Health Service in Spain [55,56]. A summary of the values and references for the parameters sourced for the model are shown in Table 2.

## 3. Results

The total number of people vaccinated in the model with aQIV or HD-QIV in the simulation was 5,126,343, based on the coverage and population shown above. The rest of the cohort remained unvaccinated. The results show that using aQIV instead of HD-QIV results in a reduction of 5405 symptomatic cases. This includes 760 primary care visits, 171 emergency room visits, 442 hospitalizations, and 26 deaths.

Incremental costs and outcomes are shown in Table 3. The incremental costs for the clinical events are lower for aQIV compared with HD-QIV. From a direct medical payer perspective, using aQIV results in a net saving of EUR 63.6 million and from a societal perspective EUR 64.2 million.

HD-QIV is dominated by aQIV as it is both more expensive and less effective, from both the societal and direct medical payer perspective. Whilst there are small savings associated with the reduction in clinical event and productivity loses the overwhelming driver is the difference in vaccine costs, which result in a net saving of EUR 61.5 million.

A series of scenario analyses were run to test the impact of the model assumptions on the ICER. These are shown in Table 4.

**Figure 3:**
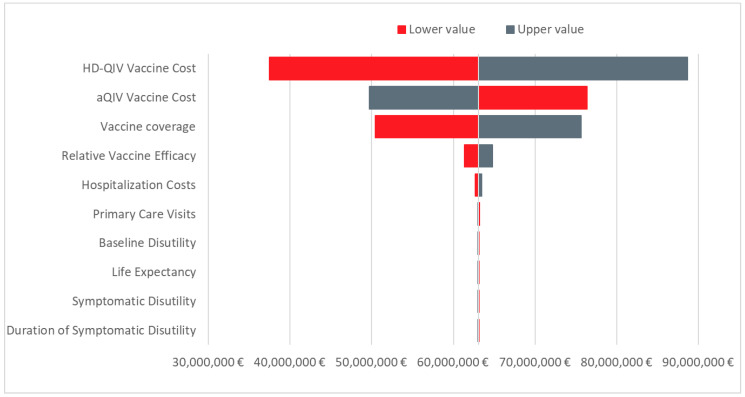
Tornado diagram showing the incremental net monetary benefit for aQIV vs. HD-QIV at a willingness-to-pay threshold of EUR 25,000 per QALY. Abbreviations: aQIV = adjuvanted QIV; HD-QIV = high-dose QIV.

A DSA was conducted with the tornado plot summarizing the 10 most influential parameters for the ICER presented in Figure 3. Vaccine costs are the most influential parameters in the model, followed by vaccine coverage. Other inputs have a relatively low impact on the cost-effectiveness.

**Figure 4:**
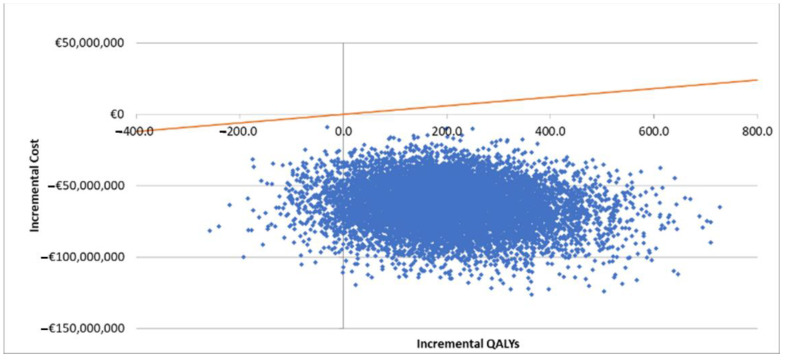
Cost-effectiveness plane for aQIV vs. HD-QIV. Abbreviation: QALY = quality-adjusted life year. Orange line represents the willingness-to-pay threshold in Spain of EUR 25,000 per QALY [55,56].

A PSA was also conducted with the scatter plots on the cost-effectiveness plane shown in Figure 4. All of the iterations fall below the EUR 25,000 per QALY willingness-to-pay threshold, which means aQIV was cost-effective in 100% of iterations. In addition, as 96% of all iterations fell within the southeast quadrant, aQIV was dominant 96% of the time.

## 4. Conclusions

Cost-effectiveness analysis is frequently used to assess the value of new vaccines, with a number of influenza models being published recently for Spain [10,23,24,57]. They enable healthcare providers to make informed decision around optimum vaccination strategies based on best available evidence. In the Spanish setting, aQIV has yet to be compared the HD-QIV in the population aged 65+. This analysis demonstrates that, largely driven by the economic benefits associated with vaccinating a large population with a less expensive vaccine with comparable effectiveness, aQIV is cost-saving compared to HD-QIV from both a direct medical payer and societal perspective. The results from Spain reflect those for the UK, Germany, and Italy, which also compared aQIV to HD-QIV [58]. Here the outcomes were considered similar with the key driver behind cost-effectiveness being cost of vaccines and the comparable effectiveness between these two enhanced vaccines.

The improvement in outcomes is driven by a non-significant improvement in vaccine effectiveness of aQIV vs. HD-QIV. This is based on a meta-analysis that involved TIV which is a limitation of the analysis. As time progresses and more data becomes available, this type of analysis can be revisited and reviewed. Moreover, the effectiveness data is being applied across all outcomes (e.g., symptomatic cases, primary care visits, emergency department visits, hospitalizations, and deaths) whilst it is primarily derived from emergency department visits and hospitalizations. This assumption is a further limitation to the analysis. However, the DSA demonstrates that these are not key drivers as the difference in vaccine costs and coverage drives the cost-effectiveness results.

Most of the data used in the analysis, such as incidence, general practitioner visits, emergency room visits, hospitalization, demographic data, resource use, costs, vaccine coverage, mortality, and some utility data are from Spain. Data from Dolk et al. [43] from the UK and Belgium though were used in previous Spanish influenza models [10]. These analyses and the estimates presented here are based on data from previously published studies. The time horizon for the model is one year, which may limit the effectiveness of the vaccine if there is cross immunity or effect across years. The assumption that the vaccine currently used in Spain is SD-QIV is conservative, as it is likely to be a mix of TIV and SD-QIV and, therefore, less effective than SD-QIV alone. Additionally, the model is static rather than dynamic, meaning herd immunity is not accounted for. However, this is a conservative assumption and likely to have no impact on the conclusions given that a small proportion of the total Spanish population is vaccinated, the number vaccinated in each cohort is the same, and vaccine effectiveness differences between the two vaccines is comparable. We also estimate incidence rates over the previous three years. Although this is an area of great uncertainty, these are unlikely to affect the conclusions, given the value drivers between the two vaccines are differences in effectiveness and price.

The impact of influenza in the population aged 65+ can be very severe and, therefore, it is vital that they are protected. There is currently a trend towards using more effective QIV vaccines that are currently replacing TIV and SD-QIV. These results should be considered during the local regional tenders in the Spanish regions, especially to provide improved healthcare with considerable savings. Given the ever-present pressures on health care budgets, aQIV offers both an affordable and cost-saving alternative to HD-QIV for this relevant group in Spain.

## Figures and Tables

**Table 1:** Rates of different clinical events per 100,000 population aged 65+.

Season	Symptomatic Cases	Primary Care Visits	Emergency Department Visits	Hospitalizations	Deaths
2017–2018	22,530	950	213	668	40
2018–2019	13,697	545	122	489	29
2019–2020	10,636	445	100	324	19

**Table 2:** Summary of parameters sourced for the model.

Parameter	Value	Source
Percentage of 65+ population vaccinated	54.7%	[17]
Life expectancy for 65+ population	9.8 years	[18]
65+ population size	9,371,743	[19]
aQIV tender price	EUR 13	[44]
aQIV list price	EUR 23	[45]
HD-QIV tender price	EUR 25	[44]
HD-QIV list price	EUR 32	[45]
Primary-care physician visits cost	EUR 59	[46,47,48]
Emergency department visit cost	EUR 183	[46,47,48]
Hospitalization cost	EUR 4467	[47,49]
Comedication cost	EUR 3.21	[50]
Probability of being employed (65–69 years old)	1.2%	[52]
Probability of being employed (75+ years old)	0.3%	[52]
Productivity loss per hour	EUR 17.44	[53]
Probability of requiring care at home (65–69 years old)	5.4%	[54]
Probability of requiring care at home (75+ years old)	14%	[54]
Baseline utility for 65+	0.65	[23]
Disutility value for symptomatic patients	0.32	[43]
Disutility value for outpatients	0.33	[42]
Disutility value for inpatients	0.6	[42]
Disutility duration for symptomatic patients	7 days	[43]
Disutility duration for outpatients	7 days	[42]
Disutility duration for inpatients	21 days	[42]
Hospital mortality rate for 65+ population	6%	[24]
Discount rates for costs and outcomes	3%	[16]

**Table 3:** Total and incremental costs and outcomes associated with aQIV and HD-QIV when used to vaccine people aged 65+ in Spain.

Category	Clinical Events			Costs (EUR Millions)		
Vaccine	HD-QIV	aQIV	Difference	HD-QIV	aQIV	Difference
Primary care visits	54,946	54,186	−760	3.42	3.37	−0.05
Emergency department visits	12,332	12,161	−171	2.26	2.23	−0.03
Hospitalization	47,371	46,930	−442	212.7	210.7	−1.98
Deaths	2842	2816	−26	0.0	0.0	0.0
Vaccine cost *				261.1	199.6	−61.5
Productivity loss				60.7	60.1	−0.6
QALY loss	21,040	20,833	−206			
LYs lost	27,940	27,679	−260			
Total costs (public payer)				479.5	415.9	−63.6
Total costs (societal)				540.2	476.0	−64.2

* Excludes administration costs which are equal across both vaccines. Abbreviations: aQIV = advanced QIV; HD-QIV = high-dose QIV; LY = life year; QALY = quality-adjusted life year.

**Table 4:** Scenario analysis for aQIV compared to HD-QIV.

Parameter	Parameter Change	Original Value	QALY Gain	Incremental Costs	ICER	Reference
Base Case			206	EUR –63.6 million	aQIV dominates HD-QIV	
Lower95% rVE	−0.05%	4.00%	−2.6	EUR –61.5 million	EUR 23,875,227 *	Figure 2
Upper95% rVE	8.40%	4.00%	433	EUR –65.8 million	aQIV dominates HD-QIV	Figure 2
Colean et al. rVE	3.20%	4.00%	165	EUR –63.2 million	aQIV dominates HD-QIV	Coleman et al. [27]
List prices	EUR 32 for HD-QIV and EUR 25 for aQIV	EUR 25 for HD-QIV and EUR 13 for aQIV	206	EUR –37.9 million	aQIV dominates HD-QIV	Vademecum [45]

* ICER for HD-QIV vs. aQIV. HD-QIV is not cost-effective vs. aQIV. Abbreviations: aQIV = advanced QIV; HD-QIV = high-dose QIV; ICER = incremental cost-effectiveness ratio; QALY = quality-adjusted life year; rVE = relative vaccine efficacy.

## Supplementary Materials

The following are available online at https://www.mdpi.com/article/10.3390/vaccines10020176/s1, Table S1: Publications were identified via a previously published systematic review and meta-analysis and a subsequent structured non-systematic review. The methods and PRISMA checklist for the previously published systematic re-view can be found in the corresponding publication [27]. The PRISMA checklist below [59] corresponds to the subse-quent structured non-systematic review conducted for this study.
